# Obesity Related Coronary Microvascular Dysfunction: From Basic to Clinical Practice

**DOI:** 10.1155/2016/8173816

**Published:** 2016-03-22

**Authors:** K. Selthofer-Relatić, I. Bošnjak, A. Kibel

**Affiliations:** ^1^Department for Cardiovascular Disease, Osijek University Hospital, J. Huttlera 4, 31000 Osijek, Croatia; ^2^Department for Internal Medicine, Faculty of Medicine, University of Osijek, Cara Hadrijana 10E, 31000 Osijek, Croatia; ^3^Department for Physiology and Immunology, Faculty of Medicine, University of Osijek, Cara Hadrijana 10E, 31000 Osijek, Croatia

## Abstract

Obesity related coronary microvascular disease is a medical entity which is not yet fully elucidated. The pathophysiological basis of coronary microcirculatory dysfunction consists of a heterogeneous group of disorders with individual morphologic/functional/clinical presentation and prognosis. Coronary microcirculatory changes include mechanisms connected with vascular dysfunction, as well as extravascular and vasostructural changes in responses to neural, mechanical, and metabolic factors. Cardiometabolic changes that include obesity, dyslipidemia, diabetes mellitus type II, and hypertension are associated with atherosclerosis of epicardial coronary arteries and/or microvascular coronary dysfunction, with incompletely understood underlying mechanisms. In obesity, microvascular disease is mediated via adipokines/cytokines causing chronic, subclinical inflammation with (a) reduced NO-mediated dilatation, (b) changed endothelial- and smooth muscle-dependent vasoregulating mechanisms, (c) altered vasomotor control with increased sympathetic activity, and (d) obesity related hypertension with cardiomyocytes hypertrophy and impaired cardiac vascular adaptation to metabolic needs. From a clinical point of view it can present itself in acute or chronic form with different prognosis, as a practice problem for real-life diagnosis and treatment.

## 1. Introduction

Obesity is a direct or indirect risk factor for cardiovascular diseases and complications contributing to morbidity and mortality. The prevalence of metabolic syndrome with visceral type of obesity exceeds 30% in the western European region as well as in the United States. In the background of cardiovascular complications, disorders of microcirculation and endothelial dysfunctions precede atherosclerosis [1]. Obese patients have ischemic signs even in the absence of obstructive/nonobstructive coronary artery disease (CAD) [2]. Patients with diabetes mellitus type II and obesity have greater risk for coronary microvascular dysfunction (CMD) than hypertensive patients [3–5]. Physiologically, adipose tissue constitutes 18–24% of total body weight while in an obese person it constitutes 52–74% [6], which cannot stand without consequential hemodynamic, metabolic, and endocrinological responses in heart morphology and function which also acts as an endocrine and immunoregulatory organ [7].

Coronary circulation commences from the aorta where oxygenated blood flows into the main right and main left coronary arteries and then branches into smaller arteries, arterioles, capillaries, venules, and the veins. These vessels network begins in the epicardium and penetrates to myocardium where coronary microcirculation includes vessels with diameters below 300 *µ*m and represents the “business end” of coronary circulation, where coronary arterial flow is exclusively diastolic and venous outflow is systolic [8, 9]. Coronary microvessel function has a pivotal role in blood pool reserve, because an organized regulation of matching local blood flow with myocardial energy demands via coronary flow conductance regulation and substance transport is crucial [10, 11]. Complex regulating mechanisms of coronary microcirculation are related to vascular functions (myogenic reactivity, flow-induced intrinsic vascular controls, response during metabolic stimulation, autoregulation, and neurohumoral mediators) and to individual heterogenous microvascular response which is orchestrated for optimal perfusion by synergistic and competitive interactions [9, 11].

According to previous studies, dysfunction of coronary microcirculation or cardiac syndrome X is defined as reduced coronary flow reserve and/or endothelial dysfunction, presented with typical angina in absence of other myocardial/cardiovascular or systemic diseases, with electrocardiographic ischemic changes and normal/minimally changed coronarogram [7, 12, 13]. The main pathophysiologic mechanism of CMD is endothelial dysfunction with impaired vasodilatation, coagulation, inflammation, permeability, cell adhesion, and altered microvascular response [14–16]. The role of obesity in CMD remains poorly understood from both the basic and clinical points of view. Obesity presents a chronic, low grade vascular inflammation, caused by macrophage infiltration, increased level of proinflammatory adipokines (leptin, resistin) and cytokines levels (IL-6, TNF alpha), and reduced levels of protective adiponectin [2]. In clinical practice there are limitations for morphologic and functional visualization of coronary microcirculation* in vivo* with standard diagnostic methods [16, 17].

## 2. Basic Aspect

### 2.1. Association of Obesity with Coronary Microvascular Function

Although obesity may affect the heart through development of other risk factors, such as dyslipidemia, glucose intolerance, insulin resistance, and proinflammatory and/or prothrombotic states, and through various potential unrecognized mechanisms, the effect of obesity on vascular function in the coronary vascular bed must be taken into account as a key (but insufficiently understood) pathogenetic factor [18]. Obesity leads to insulin resistance, vascular oxidative stress, reduced availability of vascular nitric oxide, endothelial dysfunction, and vasomotor dysfunction of the coronary microcirculation contributing to altered regulation of tissue perfusion and predisposing patients to myocardial ischemia [18–20].

Obesity has been associated with changes in coronary vascular function in animal models and in research on human subjects. In Sprague-Dawley rats fed with a high fat diet (from an age of 10 weeks, with diet composed of 24 g% fat, 24 g% protein, and 41 g% carbohydrate), there were observable decreases in acetylcholine-induced relaxation in isolated coronary microvessels after 16, 24, and 32 weeks of high fat diet and the diet also led to an impaired relaxation of aortic rings to acetylcholine, but after a period of 32 weeks. The diet led to a slow and modest increase in weight along with insulin resistance, increased free fatty acids, cholesterol, and indices of reactive oxygen species. Reversal of the high fat diet for 8 weeks, although resulting in partial recovery of metabolic parameters, failed to reverse the attenuated responses to acetylcholine [18]. Coronary relaxation responses to the potent vasodilator* calcitonin gene related peptide*, which plays a role in cardiovascular homeostasis, were found to be attenuated after 32 weeks of the same high fat diet, with an improvement of relaxation after reversal of the diet [18]. Zucker obese and Zucker diabetic fatty rats, which have improper encoding of the leptin receptor gene (manifesting as an impaired satiety reflex, with significant obesity), show progressive impairment of acetylcholine-induced relaxation of coronary microvessels (preceding changes in the aorta), as demonstrated by Oltman et al. There is an improvement when vessels of such rats are incubated with Tiron (a nonspecific free radical scavenger), suggesting reactive oxygen species as a mechanism of endothelial dysfunction [21]. Considering the work of Oltman et al., it seems that in these animal models diabetes enhances the progression of coronary vascular dysfunction, with indicators of oxidative stress preceding the development of dysfunction and possibly serving as markers of endothelial damage [21]. However, there are also studies that failed to find impaired coronary vasomotor control in prediabetic obese Zucker rats (using videomicroscopic techniques) and even measured enhanced dilation to acetylcholine and reduced vasoconstriction to endothelin [22], which makes confident conclusions about changes of coronary microvascular function in Zucker rats difficult. Research of Feher et al. showed no significant difference in the magnitude of acetylcholine-induced, endothelium-derived hyperpolarizing factor-mediated dilations in coronary arterioles isolated from lean and obese (high fat diet-fed Wistar) rats (Wistar rats on a diet with 60% of saturated fat, 58Y1, TestDiet, PMI Nutrition, fed for 10 weeks) [23]. It has been suggested that coronary microvessels are more resistant to the development of vasomotor dysfunction compared to peripheral vascular beds, having either efficient mechanisms protecting their vasomotor function or mechanisms that can actively compensate for the loss of vasomotor pathways as metabolic disease progresses [24]. The integration of vasomotor regulatory systems (myogenic, flow, or metabolic regulation of vascular resistance) in the coronary circulation is probably advantageous in conditions such as obesity and metabolic syndrome, in which metabolic and hemodynamic changes require adaptation of coronary vasomotor regulation, with a decline in adaptive vasomotor responses as the metabolic syndrome progresses [24].

Martin et al. conducted PET studies of myocardial blood flow in postmenopausal women (it was measured at rest, after the cold pressor test, and after adenosine infusion in order to determine baseline and endothelium-dependent and endothelium-independent flows). Increased waist and hip circumference, weight, and frequent weight swings were associated with impaired resting and endothelium-dependent myocardial blood flow in these human subjects [25]; it was found that the cold pressor test-induced change in endothelium-related myocardial blood flow response progressively declined in overweight and obese groups of human subjects (without arterial hypertension, smoking, and diabetes mellitus) when compared with the control group, with myocardial blood flow changes being similar in obese and morbidly obese subjects [26]. Dipyridamole-stimulated hyperemic myocardial blood flow was significantly lower in overweight, obese, and morbidly obese subjects than in human control subjects [26]. Studies on isolated pressurized coronary arterioles from patients who underwent cardiac surgery determined reduced dilations to bradykinin and the NO-donor sodium nitroprusside in obese subjects (who were normotensive). Interestingly, in subjects who were obese and hypertensive, these dilations were enhanced compared to lean individuals (and positively correlated with BMI), suggesting that simultaneous presence of obesity and hypertension activates adaptive vascular mechanisms to enhance the dilator function of coronary arterial vessels (with similar results on peripheral arterial vessels) [27]. Consequently, there are suggestions that obesity, in the presence of comorbidities, might not necessarily be associated with impaired vasodilator function of coronary microvessels but contribute to maintaining their vasodilatory capacity [24]. More investigations in this area are needed to draw final conclusions.

### 2.2. Potential Mechanisms in Changes of Coronary Microvascular Function

In addition to the increase in oxidative stress that has already been mentioned, there are a number of other mechanisms through which obesity might influence coronary microvascular function. A reduced NO-mediated dilation of coronary vessels might be connected with diminished bioavailability of NO and changes in the NO-soluble guanylate cyclase-cGMP pathway [24]. In obesity, a reduced expression of caveolin-1 leads to greater contribution of the Ca^2+^-activated potassium channels to endothelium-derived hyperpolarizing factor-mediated response, which could be important for maintained coronary dilation [23]. Chronic, low-level vascular inflammation in type 2 diabetes and obesity, with a number of proinflammatory cytokines such as IL-1*α*/*β*, IL-6, and TNF*α*, as well as upregulation of cyclooxygenase-2 expression can affect coronary vasomotor responses [24, 28], but their specific roles in coronary vascular homeostasis in obesity are largely unclear due to scarcity of available data thus far. Since adipocytes perform important endocrine functions by secreting various cytokines, hormones, and bioactive peptides, it is not surprising that alterations in adipokine levels are implicated in the development of vascular dysfunction in obesity [24]. They can influence endothelial- and smooth muscle-dependent vasoregulatory mechanisms. It has been shown that high levels of leptin attenuate dilation to acetylcholine in isolated coronary rings of healthy mongrel dogs. Leptin receptors are expressed in coronary arteries and coupled to pharmacological, nitric oxide-dependent vasodilation, with hyperleptinemia (which is present in obese individuals) causing endothelial dysfunction [24, 29]. Resistin (another adipokine) reduces endothelium-dependent dilation to bradykinin as well as endothelium-independent relaxation in response to sodium nitroprusside in porcine coronary arteries, with oxidative stress possibly playing an important role. This suggests that circulating adipokines have adverse effects on coronary vasodilator responses in obesity [24, 28, 30]. In obese human individuals, endothelium-related changes in myocardial blood flow (compared to control subjects) were inversely correlated with increase in endocannabinoid anandamide, but not with leptin or with CRP. On the other hand, there was a significant and positive correlation among endothelium-related changes in myocardial blood flow and elevated leptin and CRP, respectively, in morbidly obese individuals that was not observed for endocannabinoid anandamide, suggesting a difference between obesity and morbid obesity (as different entities) in affecting coronary circulatory function [26]. Obesity has also been associated with reduced coronary microvascular density in human subjects, which may contribute to lower maximal myocardial blood flow, impaired myocardial metabolism, diastolic dysfunction, and other changes [31].

### 2.3. Influence of Epicardial Adipose Tissue on Coronary Microcirculation

Some studies have determined a potentially important link between the epicardial adipose tissue (EAT) (the visceral fat tissue between the myocardium and visceral pericardium, which also reflects visceral adiposity rather than general obesity) [32] and vascular changes in the coronary microcirculation. It was found that EAT is predictive of an impaired coronary vasodilator capacity in patients without obstructive coronary artery disease [33, 34]. Increased EAT thickness (measured by cardiac CT) is associated with impaired myocardial flow reserve [33], which can be an indicator of microvascular dysfunction. Similarly, echocardiographic measurements of epicardial fat thickness demonstrated increased epicardial fat thickness to be independently associated with impaired coronary flow reserve in metabolic syndrome patients, but it was also found to be a predictor of worse coronary flow reserve even after accounting for the presence or absence of metabolic syndrome [34]. Coronary flow reserve is a surrogate marker for microvascular function [35]. It represents the index of maximal achievable myocardial blood flow relative to resting perfusion. Consequently, it depends highly on baseline perfusion, which is dictated by metabolic demands and is additionally highly affected by hemodynamic conditions, since hyperemic perfusion is governed by heart rate and coronary driving pressure [35, 36]. Bakkum et al. recently investigated the quantitative relationship between EAT and microvascular function, as measured by hybrid [^15^O]H_2_O positron emission tomography (PET)/CT imaging, in patients in whom hemodynamically significant coronary artery disease was excluded [36]. They performed noninvasive measurements of hyperemic coronary microvascular resistance, which might be a more reliable marker of coronary microvascular function. In this study, after adjustments for left ventricular mass and traditional risk factors, EAT volume was not significantly related to coronary microvascular function. Brinkley et al. also did not find an independent association between pericardial fat (as measured by computed tomography) and myocardial perfusion (measured by magnetic resonance imaging at rest and during adenosine-induced hyperemia) in adults without symptomatic cardiovascular disease [37]. It seems, therefore, that results in various studies are, at present, somewhat contradictory in regard to the link between EAT and microvascular function in human subjects without symptoms and obstructive coronary disease.

There is an equilibrium between the physiological roles of EAT, including biochemical, mechanical, and thermogenic cardioprotective properties, and potential pathological conditions when EAT can locally affect the heart and coronary arteries through vasocrine or paracrine secretion of proinflammatory cytokines [38]. EAT can, through cellular signaling, also induce changes in the underlying myocardium, contributing to the pathogenesis of cardiovascular disease [39]. It represents the true visceral fat depot around the heart, constituting about 20% of the total ventricular weight of the human heart while covering 80% of the heart's surface and producing various bioactive molecules [39]. A number of proinflammatory and proatherogenic adipocyte factors (including TNF-*α*, IL-6, IL-1*β*, MCP-1, PAI-1, nerve growth factor, resistin, leptin, and visfatin) from EAT have been found to participate in the various stages of atherogenesis (ranging from endothelial dysfunction to plaque destabilization and rupture) [39]. Decreased serum adiponectin levels have also been associated with impaired coronary flow reserve in female human subjects with normal epicardial coronary arteries [40].

## 3. Clinical Aspect

Conventional cardiometabolic risk factors such as hypertension, dyslipidemia, and diabetes mellitus type II together with visceral type of obesity make each patient's cardiometabolic risk profile unique for CAD or CMD [1, 12, 41]. All the components of the metabolic syndrome can individually impair endothelial functions [1]. The severity of the disease does not necessarily correlate with risk factors [13, 42, 43] but is progressive with ageing [1]. The open issues include the uncertainty of correlation between degree and distribution of microvessel changes with the level and number of risk factors and their duration. The risk profile of patients with CMD is similar to the patients with obstructive CAD [44] and it is unclear why some of them develop atherosclerotic epicardial CAD and the others CMD [12]. Also, the abnormality may not involve all coronary microvessels of a major coronary branch [45].

Increase in body mass requires a higher cardiac output and expanded intravascular volume for elevated metabolic changes resulting in left ventricular (LV) hypertrophy as an early adaptative mechanism. EAT volume directly correlates with LV mass which is associated with coronary microcirculatory function [37]. For cardiac adaptation in obesity, changes in coronary microcirculation that provide adequate blood flow for increased metabolic needs in obesity are crucial [14], but adaptive possibilities of microvessels are largely unknown [2] as well as the cellular mechanisms that alter myocardial perfusion, regulate arteriolar diameter, and adjust blood flow [16, 46]. Coronary microcirculation in basal myocardial condition in obese individuals is not compromised, but, with increased hemodynamic and metabolic changes, disruption can become manifest, as reported by Koller and coworkers [1]. Supporting the above is the fact that resistance of arteries increases with decrease of the diameter of arterioles in physiologic conditions, which is additionally altered in obesity [27].

Coronary anatomy and myocardial blood flow are major determinants for clinical symptoms [7]. Physiological conditions of the heart are characterized by suppression of alpha adrenergic vasoconstriction in the heart by myogenic, endothelial, or metabolic factors, as reported by Crea et al. [47].

Martin et al. showed that gender has an important impact; in postmenopausal obese woman myocardial blood flow negatively correlated with waist to hip ratio [25], but Peterson et al. showed that obese premenopausal women exhibited a higher myocardial blood flow, and there were no differences in men between lean and obese groups [48]. It is still matter of debate, whether gender truly has an impact on coronary regulation. Webb et al. reported that intracoronary infusion of testosterone may induce coronary artery dilation [49], while the ONTARGET and TRANSCEND trials reported that sex difference in cardiovascular risk showed no clear dependence on age nor that natural menopause is an associated risk factor for ischemic heart disease [50]. Although the research findings are ambiguous, it can be concluded that obesity in myocardial rest does not compromise myocardial microcirculation, but in increased needs dysfunction can be present [14]. Ageing could present additional risk factor for microvascular disease [1] where fibrosis plays an important role.

CMD can be classified according to pathophysiologic mechanisms by (a) the absence of obstructive CAD and myocardial disease, (b) the presence of myocardial diseases, (c) the presence of CAD, and (d) being caused by coronary recanalization [15]. According to Lanza, from a clinical point of view, it can be divided into (a) a stable/chronic form with good prognosis but with possible progression of symptoms, reduction in quality of life, and chronic myocardial remodeling and (b) an acute/unstable form where prognosis can be worse than previously thought with increased risk of myocardial infarction/death [2, 13]. CMD is associated with left ventricular systolic and diastolic contractile dysfunction in humans and animal models [51]. Rubinshtain reported that CMD is associated with a 2.5% annual major adverse rate event that includes death, nonfatal myocardial infarction, nonfatal stroke, and congestive heart failure [52].

According to previous reports, there are a number of diagnostic methods with possible clinical applications, but most of them do not meet all the parameters required for simple, risk-free, and objective clinical use. The functional status of the coronary microcirculation can be assessed by testing endothelium-dependent and endothelium-independent vascular responses [53]. PET permits functional status of coronary microcirculation and treatment effect [54]. Coronary flow reserve measured by transthoracic Doppler echocardiography is widely used but cannot distinguish between epicardial and microvascular resistance [16].

Treatment options of obesity related vascular dysfunction are limited; the most important is lifestyle change with aerobic activity, adequate nutrition, and weight loss, as well as not smoking [16]. A central target of pharmacotherapy is normalization of altered microvascular coronary structure and function with drugs that affect the microvascular function (ACE inhibitors, statins, metformin, xanthine oxidase inhibitor, and hormone replacement therapy), antianginal therapy (beta blockers; calcium channel blockers; and nitrates, trimetazidine, ivabradine, and ranolazine), medications, and procedures with the effect on pain receptors (xanthine derivatives, imipramine, and spinal cord stimulation) [42, 55]. Future targets for CMD (with/without obesity) treatment are cellular mechanisms that regulate release of various adipokines and cytokines from the adipose tissue [2], endothelin receptor antagonists [56], agents that increase NO [57], the stimulation of myocardial angiogenesis, and collateralization [16]. At the moment, there is no specific pharmacological agent with anti-inflammatory effect on chronic tissue inflammation which is a result of adipocytokines production [58].

## 4. Conclusion

**Figure 1:**
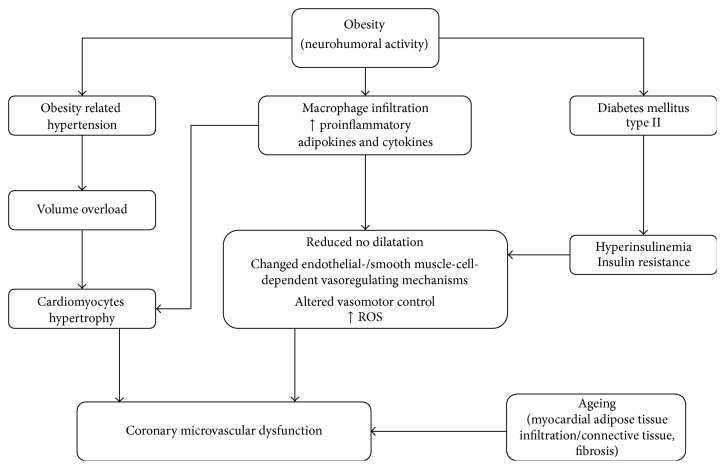
Obesity related coronary microvascular dysfunction algorithm.

Obesity is an important constituent of a whole range of risk factors for CMD, where its role is multiple: (a) it has an independent role as a pool of adipokines and cytokines with proinflammatory effects, (b) it is a part of the etiological cascade of hypertension development with volume overload and cardiomyocytes hypertrophy, and (c) it is a cause of diabetes mellitus type II development with hyperinsulinemia and insulin resistance (Figure 1). Effects of obesity on vascular reactivity, remodeling, or angiogenesis are still not fully explained, but the role of adipocytes and adipocytokines cannot be ruled out. There are certain unanswered questions, like the impact of adiposity duration, the type and degree of obesity, metabolic activity of adipose tissue, adipose tissue microcirculation, and the effect of epicardial and intramyocardial adipose tissue on coronary microcirculation. Further investigations with both basic and clinical approaches to coronary microcirculation are needed.
